# MiR-378a-3p acts as a tumor suppressor in gastric cancer *via* directly targeting RAB31 and inhibiting the Hedgehog pathway proteins GLI1/2

**DOI:** 10.20892/j.issn.2095-3941.2022.0337

**Published:** 2022-10-18

**Authors:** Xinxin Xu, Yang Li, Guoxiao Liu, Kai Li, Peng Chen, Yunhe Gao, Wenquan Liang, Hongqing Xi, Xinxin Wang, Bo Wei, Hongtao Li, Lin Chen

**Affiliations:** 1 Medical School of Chinese PLA, Beijing 100853, China; 2 Senior Department of General Surgery, The First Medical Center, Chinese PLA General Hospital, Beijing 100853, China; 3 Department of General Surgery, The 940th Hospital of Joint Logistics Support Force of People’s Liberation Army, Lanzhou 730050, China

**Keywords:** Gastric cancer, RAB31, miR-378a-3p, Hedgehog, cancer stem cells

## Abstract

**Objective:**

To improve the prognosis of patients with gastric cancer (GC), more effective therapeutic targets are urgently needed. Increasing evidence indicates that miRNAs are involved in the progression of various tumors, and RAS-associated protein in the brain 31 (RAB31) is upregulated and promotes the progression of multiple malignant tumors. Here, we focused on identifying RAB31-targeted miRNAs and elucidating their potential mechanism in the progression of GC.

**Methods:**

RAB31 and miR-378a-3p expression levels were detected in paired fresh GC tissues and GC cell lines. Bioinformatics analysis was used to predict the miRNAs targeting RAB31 and the relationships between RAB31 and other genes. Dual-luciferase reporter assays were applied to verify the targeted interaction relationship. CCK-8, colony formation, flow cytometry, wound healing, and Transwell assays were performed to assess the proliferation, apoptosis, migration, and invasion of GC cells. Tumorsphere formation assays were performed to assess the stemness of gastric cancer stem cells. Related proteins were detected by Western blot. Xenograft assays in nude mice were performed to explore the effect of miR-378a-3p *in vivo*.

**Results:**

We report the first evidence that miR-378a-3p is downregulated in GC, whereas its overexpression inhibits proliferation, invasion, and migration as well as promotes apoptosis in GC cells. Mechanistically, miR-378a-3p inhibits the progression of GC by directly targeting RAB31. Restoring RAB31 expression partially offsets the inhibitory effect of miR-378a-3p. Further research revealed that miR-378a-3p inhibits GLI1/2 in the Hedgehog signaling pathway and attenuates the stemness of gastric cancer stem cells. Finally, xenograft assays showed that miR-378a-3p inhibits GC tumorigenesis *in vivo*.

**Conclusions:**

MiR-378a-3p inhibits GC progression by directly targeting RAB31 and inhibiting the Hedgehog signaling pathway proteins GLI1/2.

## Introduction

Gastric cancer (GC) is one of the most common malignant tumors worldwide, with the 5th highest incidence rate and 4th highest mortality rate^1^. The burden of GC is expected to continue to increase^2^. With progress in diagnosis and treatment technology, the incidence and mortality of GC have decreased worldwide, but the prognosis of patients with GC remains poor. Therefore, identification of new potential targets for the diagnosis and treatment of GC is urgently needed.

MicroRNAs (miRNAs) are a class of short (20–24 nt) noncoding RNAs that posttranscriptionally regulate gene expression in multicellular organisms by affecting the stability and translation of target gene mRNAs. MiRNAs play important roles in the human metabolism, cell proliferation, apoptosis, migration, invasion, stemness, and differentiation^3^. Accumulating evidence suggests that miRNAs play critical roles in the development of multiple cancers^4–7^, and the abnormal expression of miRNAs has been detected in various malignant tumors. For example, miR-107 inhibits the proliferation of GC cells by targeting TRIAP1^8^; miRNA-543 promotes cell migration and invasion by targeting SPOP in GC^9^; and miR-18 facilitates the stemness of GC by downregulating HMGB3 through targeting Meis2^10^. Therefore, miRNAs associated with tumorigenesis and development may serve not only as biomarkers for clinical diagnosis but also as effective targets for tumor therapy.

RAB31, also known as RAB22B^11^, belongs to the small GTP-binding proteins of the RAB family^12^. In 1996, RAB31 was first isolated from melanoma cells and was found to have significant sequence similarity with RAB22. Similar to the other members of the RAB family, RAB31 is expressed throughout cells. Initially, RAB31 was found to be involved in the transport of several substances, such as that *via* glucose transporter type 4, epidermal growth factor receptor, and mannose-6-phosphate receptor^13–16^. More recently, RAB31 has been found to play an important role in human cancers, such as breast cancer^17^, glioblastoma^18,19^, and hepatocellular carcinoma^20^. For example, in breast cancer, RAB31 was first identified as an independent prognostic factor and was later found to promote cancer progression and subsequent tamoxifen resistance in patients^21–23^. Because RAB31 plays an oncogenic role in a variety of human cancers, interventions targeting RAB31 expression may aid in inhibiting the progression of GC, and using RAB31-targeted miRNAs may provide a promising method for GC treatment.

The Hedgehog-glioma-associated oncogene homolog (GLI) signaling pathway is highly conserved in mammals, and plays crucial roles in the initiation, progression, and even the stemness of multiple cancers^24^. The GLI transcription factors (GLI1, GLI2, and GLI3) are effectors of the Hedgehog signaling pathway (HHSP). Many studies have reported that GLI1 and GLI2 are involved in the occurrence, development, and stemness of GC^25–27^. Furthermore, the RAB family is involved in regulating tumor progression through the HHSP-GLI signaling pathway^28,29^.

Here, we provide the first evidence of the role of miR-378a-3p in directly targeting RAB31 in GC. First, we found that RAB31 was highly expressed, and that miR-378a-3p was downregulated, in GC tissues compared with normal tissues. Subsequently, we found that overexpression of miR-378a-3p inhibited GC progression both *in vitro* and *in vivo*. Moreover, our findings also suggested that miR-378a-3p inhibits GLI1/2 in the HHSP and further attenuates the stemness of GC stem cells (GCSCs). Therefore, our findings may provide a new target for the diagnosis and treatment of GC.

## Materials and methods

### Patients and clinical samples

Thirty pairs of fresh human GC tissues and adjacent normal tissues were collected from patients who underwent gastrectomy at the Chinese PLA General Hospital between January 2021 and April 2022. No patients received any adjuvant therapy before surgery, and their diagnoses were confirmed through postoperative pathology. The samples were collected immediately after tumor resection, kept in liquid nitrogen, and then transferred to a −80 °C refrigerator for preservation. Written informed consent was obtained from all patients. The collection of human GC tissues was approved by the Institutional Review Board of the Chinese PLA General Hospital, Beijing, China.

### Cell lines and cell culture

The immortalized gastric epithelial cell line GES-1 and 7 human GC cell lines (NCI-N87, MKN-28, BGC-823, AGS, SGC-7901, MGC-803, and HGC-27) were purchased from the Cell Bank of the Chinese Academy of Sciences (Shanghai, China). The cells were cultured in Dulbecco’s modified Eagle’s medium (DMEM, GIBCO, NY, USA) with 10% fetal bovine serum and 1% P/S in standard conditions at 37 °C and 5% CO_2_. GCSCs were obtained and cultured as previously described^30,31^. In brief, the GCSCs were cultured in ultralow-attachment 6-well plates (Corning, NY, USA) with modified DMEM/F12, 20 ng/mL epidermal growth factor (Peprotech, Hartford, CT, USA), 2% B27 supplements (Invitrogen, CA, USA), 1% insulin–transferrin–selenous acid (Corning, NY, USA), 10 ng/mL LIF (Peprotech), 10 ng/mL gastrin I (Peprotech), and 10 ng/mL basic fibroblast growth factor (Peprotech).

### Cell transfection

MiR-378a-3p mimics, inhibitor, and the corresponding negative control (NC) were purchased from JTS Biological Technology Co., Ltd. (Wuhan, China). Gain or loss of function of miR-378a-3p was accomplished by transfection of mimics, inhibitor, and the corresponding NC. The cells were seeded into 6-well plates. When the cell fusion rate reached 30%–50%, the cells were washed with 1× PBS, and 50 nM miR-378a-3p mimics or mimic NC, and 100 nM miR-378a-3p inhibitor or inhibitor NC were transfected into GC cells (BGC-823 and SGC-7901 cells) with Lipofectamine™2000 (Invitrogen). The RAB31 shRNA plasmid (target sequence: GGAGCUCAAAGUGUGCCUUTT) was synthesized by JTS Biological Technology Co., Ltd. (Wuhan, China). The wild-type RAB31 3′-UTR reporter plasmid containing miRNA binding sites and the corresponding mutant-type reporter plasmid were purchased from Promega (#E2920, USA). The plasmids were transfected as described above. All oligonucleotides and plasmids used are listed in **Supplementary Table S1**.

### RNA isolation and qRT-PCR

Total RNA was extracted from the cell lines or tissues with TRIzol reagent according to the manufacturer’s instructions (Invitrogen). The total RNA concentrations were determined with a NanoDrop 2000 spectrophotometer (Thermo Fisher, MA, USA). For the mRNA quantification, cDNA was synthesized with SweScript All-in-one First-Strand cDNA Synthesis SuperMix, and qPCR was performed with 2× Universal Blue SYBR Green qPCR Master Mix (Servicebio, Wuhan, China). For the miRNA quantification, cDNA was synthesized with a miRNA 1st Strand cDNA Synthesis Kit (by stem–loop), and miRNA Universal SYBR qPCR Master Mix (Vazyme, Nanjing, China) was used to perform qPCR. All qPCR analyses were performed on a CFX96 Real-Time PCR Detection System (Bio-Rad, CA, USA). The mRNA and miRNA expression levels were normalized to the expression levels of GAPDH and U6, respectively. The results were analyzed with the 2^−ΔΔCT^ method, and each sample was analyzed in triplicate. All primers used are listed in **Supplementary Table S2**.

### Protein extraction and Western blot analysis

Cell precipitates were collected by conventional centrifugation, and cells were lysed on ice with RIPA extraction buffer (Beyotime, Shanghai, China) containing protease inhibitors. Fifteen minutes later, the proteins were collected by centrifugation at a low temperature and high speed, and the protein quantification and concentration were determined with a BCA protein kit (Solarbio, Beijing, China). Subsequently, the protein lysates were separated by SDS–PAGE and transferred to PVDF membranes (Millipore, Darmstadt, Germany). The membranes were blocked with 5% skim milk for 2 h at room temperature and incubated with primary antibodies overnight at 4 °C. Subsequently, the membranes were incubated with HRP-conjugated secondary antibodies for 2 h at room temperature. B-Actin expression was used as an internal control for normalization. The bands were detected with enhanced ECL chemiluminescence reagent (Beyotime). The details of the antibodies used in the experiments are listed in **Supplementary Table S3**.

### Immunohistochemistry (IHC)

Paraffin-embedded patient tissues were cut into 5-μm-thick sections, dewaxed, and rehydrated with xylene and alcohol. The slides were immersed in antigenic repair solution (citric acid pH 6.0/EDTA buffer pH 8.0), and antigenic repair was performed in a microwave oven at high heat for 3 min and low heat for 5 min. After the inactivation of endogenous peroxidase, the slides were washed with PBS 3 times and blocked with 10% BSA solution at room temperature. The sections were incubated overnight with primary antibodies at 4 °C and then incubated with HRP-conjugated secondary antibodies at room temperature for 1 h. The peroxidase reaction was visualized with 3,3-diaminobenzidine. Finally, the slices were redyed with hematoxylin and photographed under a microscope, and the images were analyzed.

### Dual-luciferase reporter assays

Dual-luciferase reporter assays were used to verify the targeting relationship between miR-378a-3p and RAB31. First, the human embryonic kidney cell line HEK293T was cultured for luciferase reporter assays. When the cell fusion rate reached 40%–60%, 100 nM miR-378a-3p mimics or the corresponding NC were cotransfected with the wild-type (WT) or mutant (MUT) RAB31 3′-UTR luciferase reporter plasmids. Subsequently, a dual-luciferase assay system (#E2920, Promega, WI, USA) was used to determine relative luciferase activities according to the manufacturer’s instructions 48 h after transfection. Firefly luciferase activity was normalized by *Renilla* luciferase activity. The same procedure was repeated in GC cell lines (BGC-823 and SGC-7901 cells).

### Cell proliferation assays

The viability of treated and control BGC-823 and SGC-7901 cells was examined with Cell Counting Kit-8 (CCK-8) assays (Beyotime). Briefly, cells from each group in logarithmic growth phase were collected, resuspended, counted, and seeded into 96-well plates at a density of 3,000 cells per well. Four wells were established for each group. CCK-8 assays were performed at 0, 24, 48, 72, and 96 h after cell culture. Ten microliters of CCK-8 reagent (Solarbio) was added to each well, and the absorbance was measured at 450 nm after the cells were cultured for 1 h at 37 °C. The proliferation curves were plotted to denote the cell survival rate.

### Colony formation assays

Cells from the treatment and control groups were collected by routine centrifugation and counted. In total, 500 cells per well were seeded into 6-well plates with complete DMEM. The cells were incubated at 37 °C and 5% CO_2_ for 10–14 days until visible colonies formed. Subsequently, the cells were treated with 4% paraformaldehyde for 15 min and stained with 0.1% crystal violet for 30 min. The colonies were imaged and counted.

### Wound healing assays

The migration ability of each group of GC cells was detected with wound healing assays. Six-well plates were cultured until 90%–95% fusion of GC cells. A straight line was scratched on the single cell layer with a sterile 200-μL pipette tip, and then the plate was washed twice with PBS and photographed; the medium was changed to serum-free medium, and the cells were cultured for another 48 h. The relative migration distance of the GC cells was calculated according to the width of the scratch at 0 h and 48 h.

### Transwell assays

Cell invasion and migration assays were performed in 24-well plates with 6.5-mm diameter inserts and 8-μm pore size Transwell chambers (Corning, USA). Cell culture medium containing 20% fetal bovine serum was added to the 24-well plates at 600 μL per well. Subsequently, 200 μL of serum-free medium containing a certain number of cells was added to each upper chamber. For the migration assays, 4 × 10^4^ to 5 × 10^4^ cells in 200 μL serum-free medium were diluted and added to each chamber. For the invasion assays, each chamber was precoated with 50 μL of diluted Matrigel (Corning, Matrigel:medium = 1:9), and then 8 × 10^4^ to 10 × 10^4^ cells were diluted with 200 μL of serum-free medium and added to each chamber. After incubation at 37 °C for 24 h, the cells in the upper layer of the chambers were gently dabbed with a cotton swab. The cells at the bottoms of the chambers were fixed with paraformaldehyde for 15 min and stained with crystal violet for 30 min. The penetrated cells were photographed and counted.

### Flow cytometry

For apoptosis detection, pretreated cells cultured on 6-well plates were digested, collected, and centrifuged with trypsin free of EDTA. The cells were then washed with ice-cold PBS and resuspended in 500 μL complete DMEM. The cells were counted and diluted to 1 × 10^5^ to 3 × 10^5^ cells/mL. Subsequently, a Muse^®^ Annexin V & Dead Cell Assay (Luminex Corporation, USA) was used to detect the apoptosis rate of the GC cells. For detection of CD44 expression, the cells were suspended in 80 μL binding buffer (Beyotime) and then incubated with 2 μL PE-Cy7 CD44 primary antibody (#560533, BD Biosciences) at 4 °C for 15–20 min in the dark. The cells were resuspended in 500 μL Hanks’ balanced salt solution after centrifugation, and the expression of CD44 was detected with a BD flow cytometer. The data analysis was performed in FlowJo™ software (Version 10, Tree Star, USA). All steps were performed in accordance with the requirements of the protocol.

### Xenograft assays

BALB/c nude mice (male, 5 weeks old, 16–20 g) were purchased from Vital River Laboratory Animal Co. (Beijing, China) and raised under specific-pathogen-free conditions. Twelve nude mice were randomly divided into the miR-378a-3p mimic NC group and mimic group (6 mice per group). Subsequently, 1 × 10^6^ BGC-823 cells were transfected with mimic NC or mimics mixed with Matrigel (Corning, America) at a 1:1 ratio. Subsequently, the above mixture was injected subcutaneously into the right flanks of nude mice to establish the xenograft tumor model. Tumor size was measured and recorded every 4 to 5 days with Vernier calipers. Three weeks later, the mice were sacrificed. Subsequently, the tumors were dissected and measured. The tumor volume was calculated with the following formula: V = 1/2 × length × width^2^. Animal experiments conformed to the standards set by the Declaration of Helsinki, and the guidelines were approved by the Laboratory Animal Ethics Committee of the Chinese PLA General Hospital.

### Bioinformatics analysis

The RAB31-targeted miRNAs were predicted with the online databases TargetScan (http://www.targetscan.org/vert_72/), DIANA (http://diana.imis.athena-innovation.gr/DianaTools/index.php), mirDIP (http://ophid.utoronto.ca/mirDIP/index.jsp#r), and StarBase (http://starbase.sysu.edu.cn/index.php). The intersection of the predicted results was obtained with an online Venn diagram (https://bioinfogp.cnb.csic.es/tools/venny/). The Cancer Genome Atlas (TCGA) (https://www.cancer.gov/about-nci/organization/ccg/research/structural-genomics/tcga) and the Gene Expression Profiling Interactive Analysis (GEPIA) (http://gepia.cancer-pku.cn/) database were used to analyze the RAB31 expression levels in GC and adjacent normal tissues. The online database Kaplan–Meier plotter (http://kmplot.com/analysis/index.php) was used for survival analysis. The expression levels of miRNAs, their correlations with those of target genes, and the correlations between genes were analyzed on the basis of TCGA database.

### Statistical analysis

SPSS version 26.0 (IBM Corporation, Armonk, NY, USA) was used for the statistical analysis in this study. Experimental results that conformed to a normal distribution are expressed as mean ± standard deviation. Student’s t test (two-tailed) or analysis of variance was applied for comparisons between 2 or more groups. The correlations between 2 parameters were tested with a Pearson correlation test. GraphPad Prism 8.0 was used for data visualization. (*P < 0.05, **P < 0.01, ***P < 0.001. ns = no significance. *P* < 0.05 was considered to indicate statistically significant differences.)

## Results

### RAB31 is upregulated in GC and predicts poor prognosis

**Figure 1:**
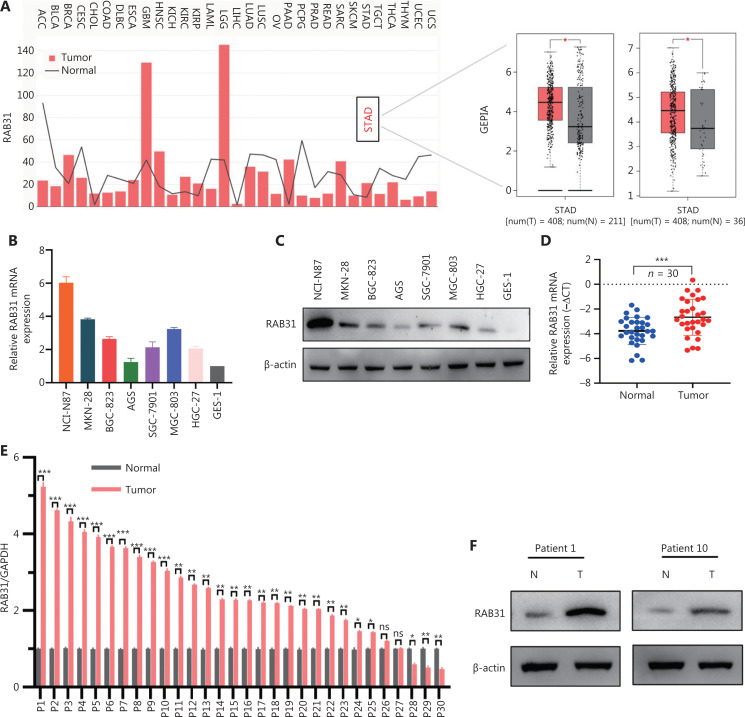

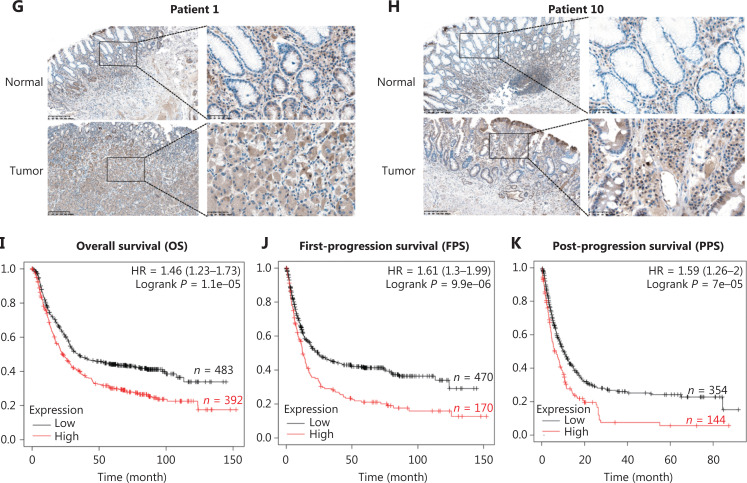
RAB31 is upregulated in GC and predicts poor prognosis. (A) Expression of RAB31 mRNA in different tumor types including gastric cancer (GC) and corresponding normal tissues, according to GEPIA and TCGA database analysis. (B, C) mRNA and protein expression of RAB31 in 7 GC cell lines and GES-1 cells. (D, E) Relative RAB31 mRNA expression in 30 pairs of GC and adjacent normal tissues. (F-H) RAB31 protein expression and immunohistochemical staining in representative patients with GC (patient 1 and patient 10). (I-K) Survival analysis *via* the K-M Plotter online database (scale bar = 200 μm, *P < 0.05, **P < 0.01, ***P < 0.001, ns = no significance).

First, we used the GEPIA and TCGA databases to investigate the expression levels of RAB31 mRNA in different tumors and corresponding normal tissues. As shown in **Figure 1A**, RAB31 was upregulated in GC tissues compared with adjacent normal tissues. In addition, in the paired and unpaired samples from the Genotype-Tissue Expression (GTEx) database, statistically significant differences were observed between the GC and para-cancer tissues (**Figure 1A**). Subsequently, to determine the expression of RAB31 in GC, we detected the expression of RAB31 mRNA in the immortalized gastric epithelial cell lines GES-1 and 7 GC cell lines. Through qRT–PCR, we found that RAB31 showed greater upregulation in most GC cell lines than GES-1 (**Figure 1B**). Then, we further detected RAB31 protein expression in GC cell lines. In accordance with the qRT–PCR results, the WB results showed that RAB31 protein was highly expressed in most GC cell lines (**Figure 1C**). Next, we detected the RAB31 mRNA levels in 30 paired gastric adenocarcinoma and adjacent normal tissues from patients with GC, and found that the difference in RAB31 expression levels between GC and adjacent normal tissues was statistically significant, and RAB31 was highly expressed in 25/30 patients (**Figure 1D and 1E**). Moreover, WB and IHC analyses were performed to determine the protein expression of RAB31 in representative patients (**Figure 1F–1H**). In addition, on the basis of the K-M Plotter online database, patients with higher RAB31 expression had a lower overall survival, first-progression survival, and post progression survival, thus indicating that high RAB31 expression predicted poor prognosis in patients with GC (**Figure 1I–1K**). Collectively, our findings indicated that RAB31 was highly expressed in GC and predicted poor prognosis.

### MiR-378a-3p is underexpressed in GC and suppresses the expression of RAB31

**Figure 2:**
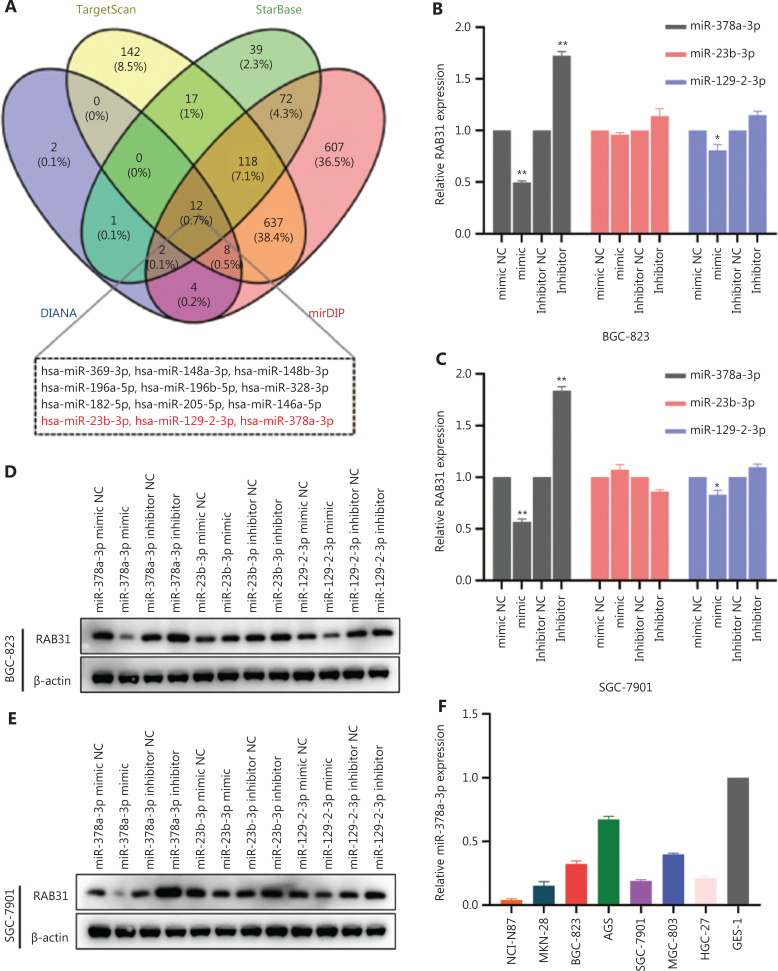

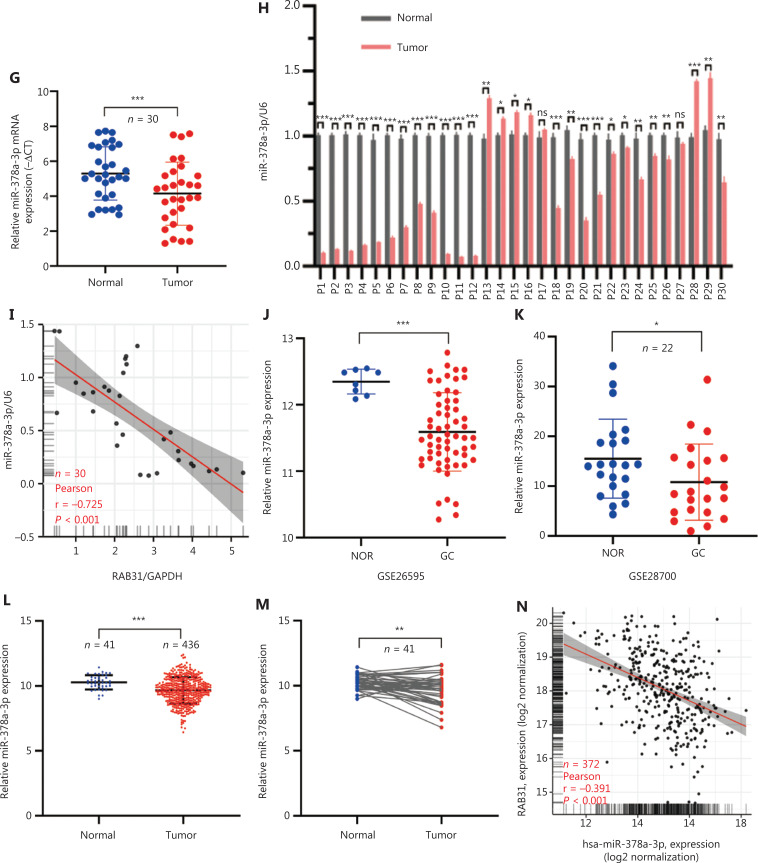
MiR-378a-3p is underexpressed in GC and downregulates the expression of RAB31. (A) Venn diagram of RAB31-targeted miRNAs, predicted by bioinformatic analysis. (B, C) RAB31 mRNA expression after transfection with 3 different candidate miRNA mimics, inhibitor, and negative control (NC) in BGC-823 and SGC-7901 cells. (D, E) Protein expression of RAB31 in BGC-823 and SGC-7901 cells after transfection with 3 different candidate miRNA mimics, inhibitor, and NC. (F) Relative miR-378a-3p expression in 7 GC cell lines and GES-1 cells. (G, H) Relative miR-378a-3p expression in 30 paired tissues from patients with GC. (I) Correlation between miR-378a-3p and RAB31 mRNA expression in 30 GC tissues. (J, K) Relative expression of miR-378a-3p in GC and normal tissues, on the basis of the GSE26595 and GES28700 datasets from the GEO database. (L) Relative expression of miR-378a-3p in GC and normal tissues in TCGA. (M) Relative expression of miR-378a-3p in 41 paired GC and adjacent normal tissues in TCGA. (N) Correlation between miR-378a-3p and RAB31 mRNA expression in GC tissues in TCGA (*n* = 372) (*P < 0.05, **P < 0.01, ***P < 0.001, ns = no significance).

Many studies have shown that miRNAs modulate gene expression^4^. Hence, to identify the potential RAB31-targeted miRNAs in GC, we used online databases including TargetScan, StarBase, DIANA, and mirDIP for bioinformatics analysis. Subsequently, we intersected the predicted results and obtained 12 candidate miRNAs. Next, we predicted the expression levels of these miRNAs in GC on the basis of the TCGA database and found that 9 miRNAs were highly expressed. After excluding these 9 miRNAs, we finally obtained 3 candidate miRNAs (miR-23b-3p, miR-129-2-3p, and miR-378a-3p) (**Figure 2A**). We then sought to determine which of these miRNAs actually function in GC. Therefore, we synthesized miRNA mimics and inhibitors of these 3 candidates and transferred them into 2 GC cell lines (BGC-823 and SGC-7901 cells). We then assessed the changes in RAB31 expression with qRT–PCR and WB. All 3 miRNAs regulated RAB31 mRNA levels to varying degrees, but miR-378a-3p had the most significant negative regulatory effect on RAB31 (**Figure 2B and 2C**). Subsequently, we performed WB experiments to verify the regulatory effects of the 3 miRNAs on RAB31 at the protein level. In agreement with the qRT–PCR results, WB indicated that miR-378a-3p had the most significant negative regulatory effect on RAB31 (**Figure 2D and 2E**). In short, the above results indicated that miR-378a-3p had the strongest ability to downregulate RAB31 expression in GC among the 3 candidate miRNAs. On the basis of these results, we sought to determine the expression level of miR-378a-3p in GC and its correlation with RAB31 mRNA. First, we quantified the expression levels of miR-378a-3p in GES-1 and 7 GC cell lines by qRT–PCR. MiR-378a-3p was more downregulated in most GC cell lines than GES-1 cells (**Figure 2F**). Subsequently, we detected the relative expression of miR-378a-3p in 30 paired GC and paracancerous normal tissues. MiR-378a-3p expression significantly differed between groups, and miR-378a-3p was downregulated in most GC tissues (22/30) (**Figure 2G and 2H**). The correlation between miR-378a-3p and RAB31 mRNA expression in GC tissues from 30 patients was analyzed, and the results showed a significant negative correlation between miR-378a-3p and RAB31 mRNA (Pearson R = −0.725, *P* < 0.001) (**Figure 2I**). Subsequently, analysis based on the GSE26595 and GES28700 datasets from the GEO database confirmed the downregulation of miR-378a-3p in GC tissues (**Figure 2J and 2K**). Furthermore, on the basis of the STAD project of TCGA, we analyzed the relative expression levels of miR-378a-3p in GC and normal adjacent tissues. Similarly, miR-378a-3p expression was significantly downregulated in GC tissues (*P* < 0.001) (**Figure 2L**). Data analysis of 41 pairs of GC and adjacent tissues on the basis of the TCGA database also showed that miR-378a-3p had low expression in cancer tissues (*P* < 0.01) (**Figure 2M**). In addition, we observed a significant negative correlation between miR-378a-3p and RAB31 mRNA in 372 TCGA GC samples (Pearson R = −0.391, *P* < 0.001) (**Figure 2N**). Overall, miR-378a-3p was underexpressed in GC, miR-378a-3p suppressed the expression of RAB31 in GC cell lines, and miR-378a-3p mRNA expression showed a significant negative correlation with RAB31 mRNA expression.

### MiR-378a-3p directly targets the 3′-UTR of RAB31 mRNA

**Figure 3:**
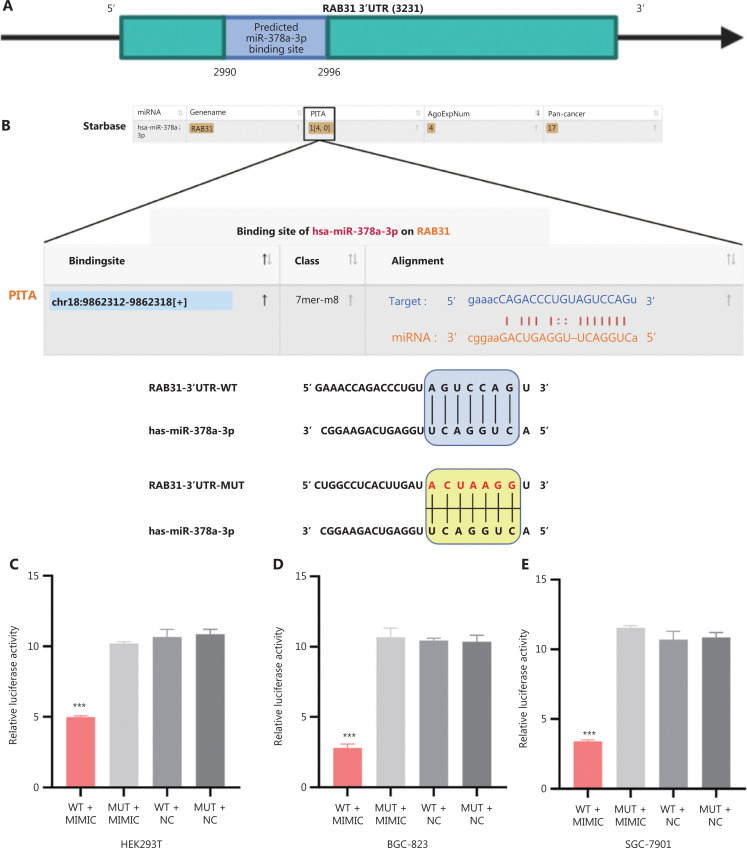
MiR-378a-3p directly targets the 3′-UTR of RAB31 mRNA. (A) Schematic diagram of the miR-378a-3p binding sites in the 3′-UTR of RAB31 mRNA, predicted by PITA. (B) Sequences of the WT-RAB31 and MUT-RAB31 3′-UTR reporter plasmids. (C-E) Relative luciferase activity in HEK293T cells and GC cell lines (BGC-823 and SGC-7901 cells) cotransfected with miR-378a-3p mimics or NC, and WT-RAB31 or MUT-RAB31 reporter plasmid (***P < 0.001).

On the basis of our results, we speculated that RAB31 mRNA may be a direct target gene of miR-378a-3p. To verify this hypothesis, we performed bioinformatics analysis to predict the miR-378a-3p binding sites of the RAB31 mRNA 3′-UTR. According to the StarBase and PITA databases, the 2990–2996 sequences of the RAB31 mRNA 3′-UTR were potential binding sites of miR-378a-3p (**Figure 3A**). Therefore, we constructed WT and MUT plasmids containing miR-378a-3p binding sites (**Figure 3B**), and performed dual-luciferase reporter assays in HEK293T cells and GC cell lines (BGC-823 and SGC-7901 cells). WT-RAB31 luciferase activity significantly decreased after cotransfection with miR-378a-3p mimics (*P* < 0.001). However, we observed no significant change in MUT-RAB31 luciferase activity after cotransfection with miR-378a-3p mimics (**Figure 3C–3E**). Collectively, the above results indicated that RAB31 is a direct target of miR-378a-3p.

### MiR-378a-3p inhibits GC progression and promotes apoptosis in GC cells

**Figure 4:**
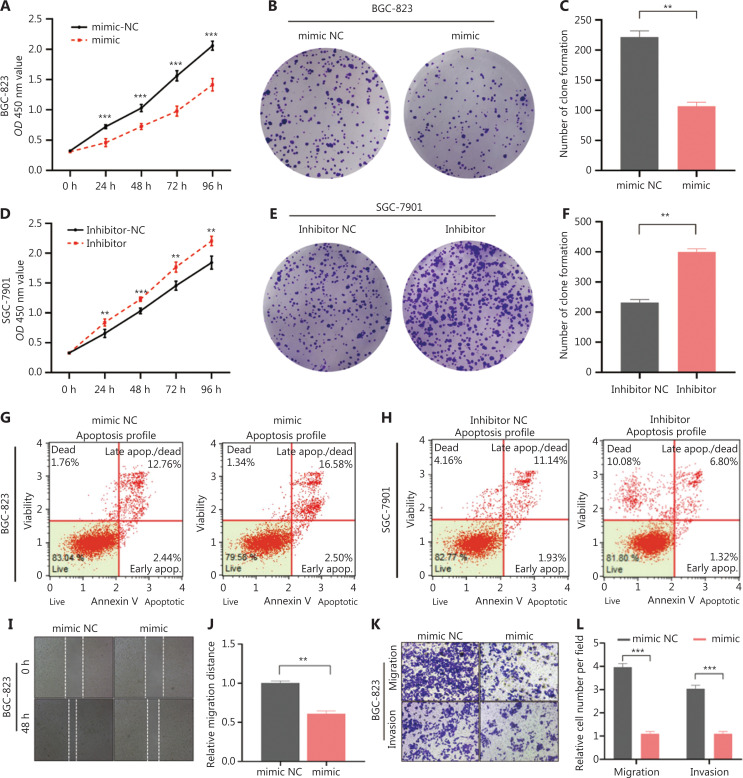

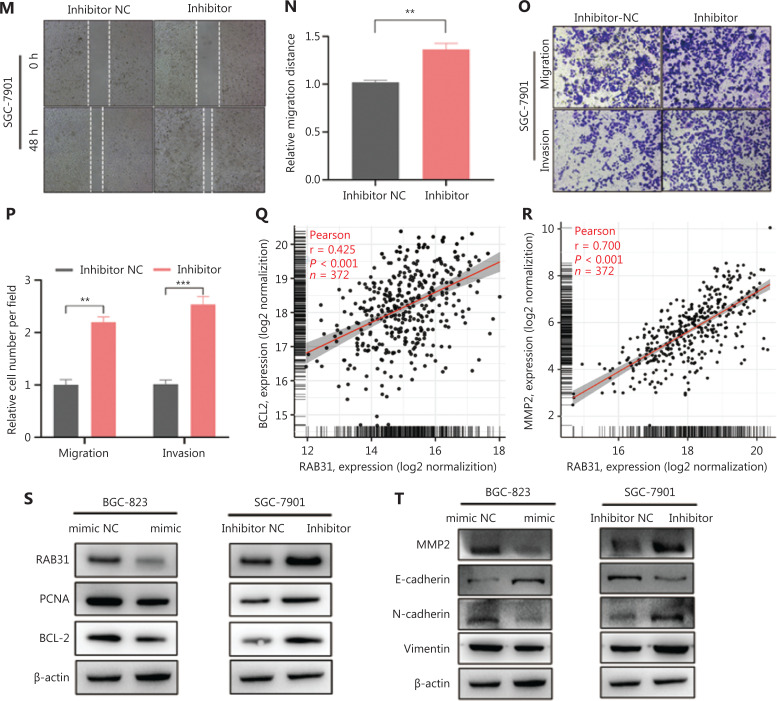
MiR-378a-3p inhibits GC progression and promotes apoptosis of GC cells. (A) CCK-8 assays indicating the viability of BGC-823 cells transfected with miR-378a-3p mimics or NC. (B, C) Colony formation assays of BGC-823 cells transfected with miR-378a-3p mimics or NC. The number of BGC-823 cell colonies and statistical analysis are shown. (D) Viability of SGC-7901 cells, detected with CCK-8 assays after transfection with miR-378a-3p inhibitor or NC. (E, F) Colony formation assays of SGC-7901 cells transfected with miR-378a-3p inhibitor or NC. The number of SGC-7901 cell colonies and statistical analysis are shown. (G) Flow cytometry detection of the apoptosis rate of BGC-823 cells transfected with miR-378a-3p mimics or NC. (H) Flow cytometry detection of the apoptosis rate of SGC-7901 cells after transfection with miR-378a-3p inhibitor or NC. (I, J) Wound healing assay detection of the migration of BGC-823 cells transfected with mimics or NC. The relative migration distance was calculated. (K, L) Transwell assay detection of the migration and invasion of BGC-823 cells transfected with mimics or NC. The migrated and invaded cells were counted. (M, N) Wound healing assay detection of the migration of SGC-7901 cells treated with inhibitor or NC. The relative migration distance was calculated. (O, P) Transwell assay detection of the migration and invasion of SGC-7901 cells treated with inhibitor or NC. The migrated and invaded cells were counted. (Q, R) Correlations among BCL-2 and RAB31, MMP2, and RAB31, analyzed on the basis of TCGA database. (S, T) Expression of proteins associated with proliferation, apoptosis, invasion, and EMT after miR-378a-3p intervention in BGC-823 and SGC-7901 cells (**P < 0.01, ***P < 0.001).

Previous studies have shown that miR-378a-3p is a critical tumor suppressor in multiple cancers, including hepatocellular carcinoma^32^, glioma^33^, and retinoblastoma^34^. However, the function and mechanism of miR-378a-3p in GC have not been thoroughly explored. Therefore, we transfected miR-378a-3p mimic and mimic NC into BGC-823 cells, and transfected miR-378a-3p inhibitor and inhibitor NC into SGC-7901 cells, to explore the function of miR-378a-3p in GC. First, we manipulated the expression of miR-378a-3p to assess its effect on the proliferation ability of GC cells. After transfection with miR-378a-3p mimics, the proliferation ability and the number of colonies formed by GC cells decreased (**Figure 4A–4C**). In contrast, the cell proliferation ability and the number of colonies formed by GC cells increased after transfection with the miR-378a-3p inhibitor (**Figure 4D–4F**). Next, the apoptosis rate of GC cells after transfection with miR-378a-3p mimics or inhibitor was detected. The apoptosis rate of GC cells after transfection with mimics *vs.* inhibitor showed opposite trends (**Figure 4G and 4H**). Subsequently, we explored the effects of miR-378a-3p expression on the invasion and migration of GC cells. The migration and invasion abilities were significantly weakened after transfection with miR-378a-3p mimics (**Figure 4I–4L**), whereas cell migration was enhanced after transfection with miR-378a-3p inhibitor (**Figure 4M–4P**). We then analyzed the correlations among RAB31 and BCL2, RAB31, and MMP2 in the TCGA database. RAB31 was positively correlated with BCL2 (Pearson R = 0.425, *P* < 0.001) and MMP2 (Pearson R = 0.700, *P* < 0.001) (**Figure 4Q and 4R**). Finally, we detected the expression of proliferation-, apoptosis-, invasion-, and epithelial mesenchymal transformation (EMT)-associated proteins after transfection with miR-378a-3p mimics and inhibitor. Almost all protein expression trends were consistent with the above results. However, E-cadherin increased after transfection with miR-378a-3p mimics (**Figure 4S and 4T**). Together, these results indicated that enhanced miR-378a-3p expression inhibits GC progression and promotes apoptosis in GC cells.

### RAB31 partially reverses miR-378a-3p-mediated suppression of GC

**Figure 5:**
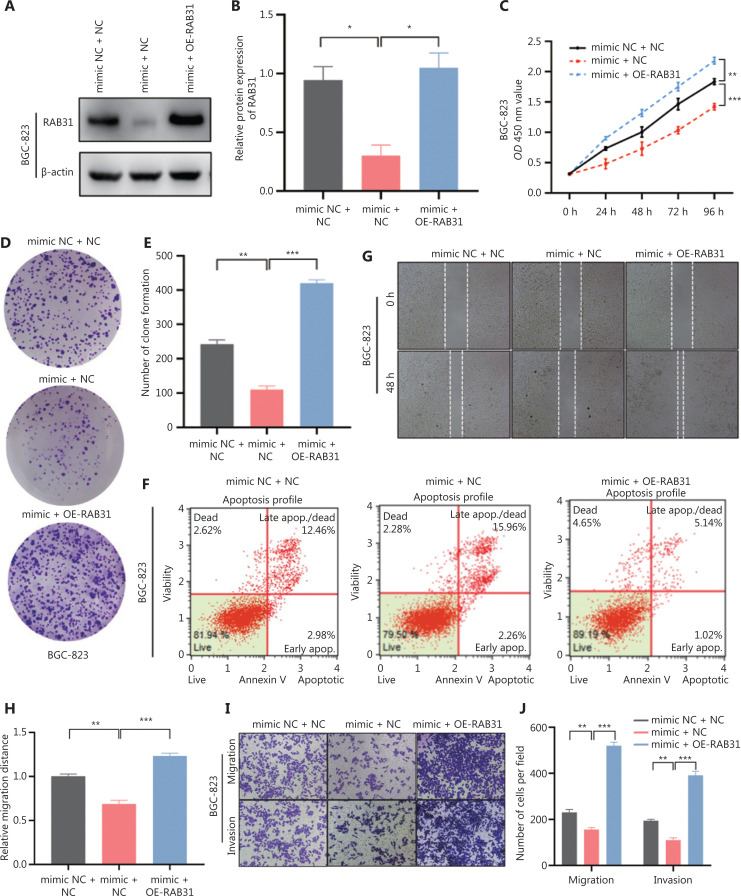

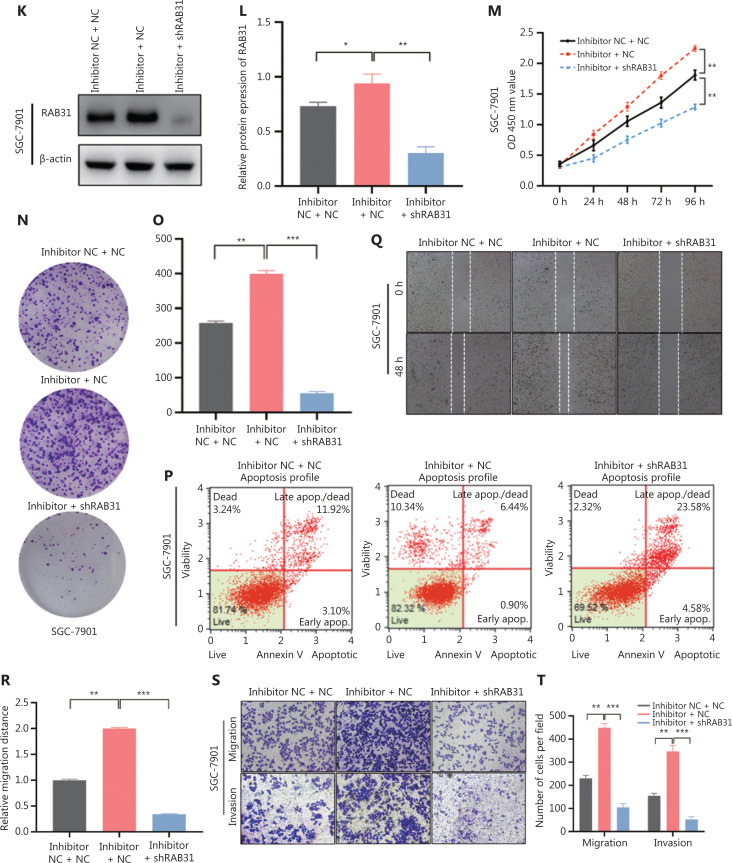
RAB31 partially reverses miR-378a-3p-mediated suppression of GC. (A, B) Protein expression and statistical analysis of RAB31 in BGC-823 cells cotransfected with miR-378a-3p mimics and RAB31 overexpression (OE-RAB31) plasmid. (C) Proliferation ability of BGC-823 cells, determined by CCK-8 assays. (D, E) Number of transfected BGC-823 cell colonies. (F) Apoptosis rate of BGC-823 cells, detected *via* flow cytometry. (G, H) Migration of treated BGC-823 cells, detected by wound healing assays. The relative migration distance was calculated. (I, J) Transwell assay detection of the migration and invasion abilities of BGC-823 cells. The cells were counted. (K, L) Protein expression and statistical analysis of RAB31 in SGC-7901 cells cotransfected with miR-378a-3p inhibitor and RAB31 shRNA (shRAB31) plasmid. (M) Proliferation ability of SGC-7901 cells, determined with CCK-8 assays. (N, O) Number of SGC-7901 cell colonies. (P) Apoptosis rate of SGC-7901 cells, measured *via* flow cytometry. (Q, R) Migration of SGC-7901 cells, detected by wound healing assays. The relative migration distance was calculated. (S, T) Transwell assay detection of the migration and invasion abilities of SGC-7901 cells. The cells were counted (*P < 0.05, **P < 0.01, ***P < 0.001).

Our previous findings confirmed that miR-378a-3p downregulated the expression of RAB31, inhibited the progression of GC, and promoted apoptosis of GC cells. Nevertheless, whether miR-378a-3p inhibits GC progression by regulating RAB31 remained unclear. To test this possibility, we performed rescue experiments. First, we restored the expression of RAB31 in BGC-823 cells transfected with miR-378a-3p mimics, and observed that the tumor suppressive effects of miR-378a-3p were partially reversed (**Figure 5A–5J**). Subsequently, we knocked down RAB31 expression in SGC-7901 cells transfected with miR-378a-3p inhibitor and also observed that knockdown of RAB31 offset the cancer-promoting effect of the miR-378a-3p inhibitor (**Figure 5K–5T**). In conclusion, these results indicated that miR-378a-3p inhibits GC progression by regulating RAB31.

### MiR-378a-3p inhibits GLI1/2 expression and further inhibits GC stemness

**Figure 6:**
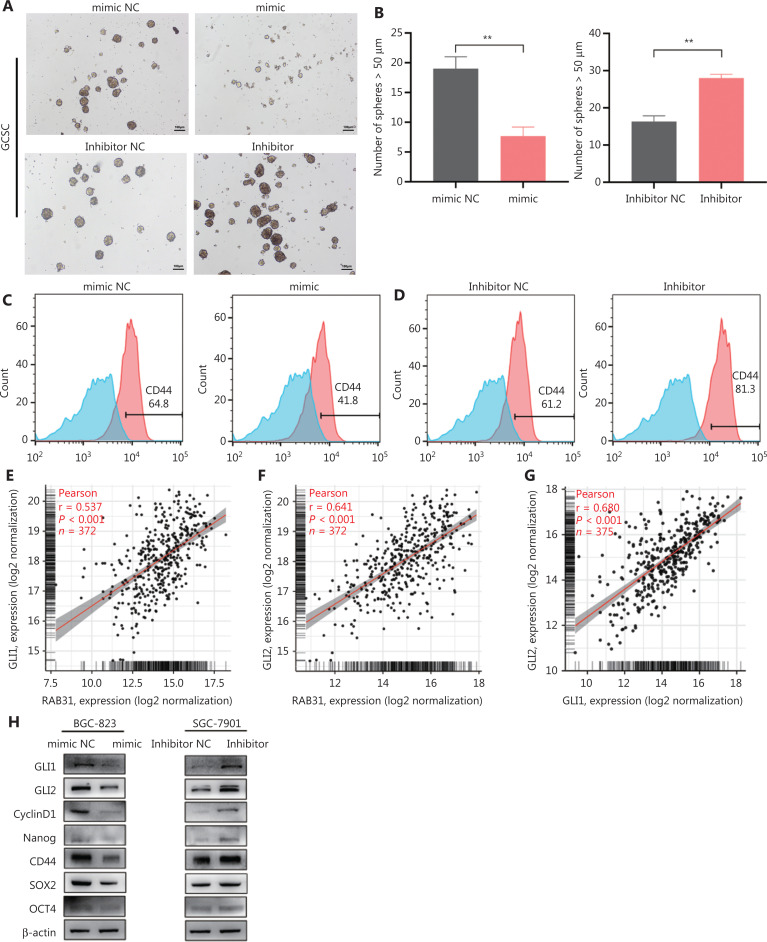
MiR-378a-3p inhibits GLI1/2 and GC stemness. (A, B) Tumorsphere formation assays of gastric cancer stem cells (GCSCs) transfected with miR-378a-3p mimics or inhibitor and the corresponding NC. The number of tumorspheres >50 μm per field was counted and statistically analyzed. (C, D) Expression of CD44 after miR-378a-3p treatment in GCSCs, detected by flow cytometry. (E-G) Correlations between GLI1 and RAB31, GLI2 and RAB31, and GLI1 and GLI2, analyzed on the basis of TCGA database. (H) Expression of GLI1, GLI2, Cyclin D1, and stemness-associated proteins after miR-378a-3p treatment in BGC-823 and SGC-7901 cells (scale bar = 100 μm, **P < 0.01).

GLI1 and GLI2, key molecules in the HHSP, have been reported to play important roles in maintaining the stemness of GC cells^35–38^. Therefore, we wondered whether abnormally expressed miR-378a-3p might inhibit GLI1/2 expression and further influence the stemness of GC cells. First, GCSCs were cultured, and miR-378a-3p mimics or inhibitor was transfected into these cells. As expected, the tumorsphere formation ability of GCSCs was significantly attenuated after transfection with miR-378a-3p mimics, whereas the tumorsphere formation ability was enhanced after transfection with miR-378a-3p inhibitor (**Figure 6A and 6B**). Second, we performed flow cytometry to detect the expression of CD44 on the surfaces of GCSCs. The results confirmed that the expression of CD44 in GCSCs significantly decreased after transfection with miR-378a-3p mimics, whereas opposite results were observed after transfection with miR-378a-3p inhibitor (**Figure 6C and 6D**). Furthermore, on the basis of the STAD project of the TCGA database, we analyzed the correlations among RAB31, GLI1, and GLI2. The results indicated a significant positive correlation between RAB31 and GLI1 mRNA (Pearson R = 0.537, *P* < 0.001), RAB31 and GLI2 mRNA (Pearson R = 0.641, *P* < 0.001), and GLI1 and GLI2 mRNA (Pearson R = 0.680, *P* < 0.001) (**Figure 6E–6G**). Because no direct targeting site between miR-378a-3p and the GLI 1/2 3′-UTR exists, we reasoned that miR-378a-3p might attenuate the stemness of GCSCs by inhibiting GLI1/2, perhaps partially through targeting RAB31. To test this hypothesis, we examined the changes in GLI1, GLI2, a hedgehog target gene (cyclin D1)^39^, and stemness-associated proteins through WB in BGC-823 and SGC-7901 cells after transfection with miR-378a-3p mimics or inhibitor. MiR-378a-3p inhibited GLI1/2 in the hedgehog pathway and attenuated the stemness of GCSCs (**Figure 6H**). Together, these results showed that miR-378a-3p overexpression inhibits GLI1/2 and further inhibits GC stemness.

### MiR-378a-3p inhibits tumorigenesis in vivo

**Figure 7:**
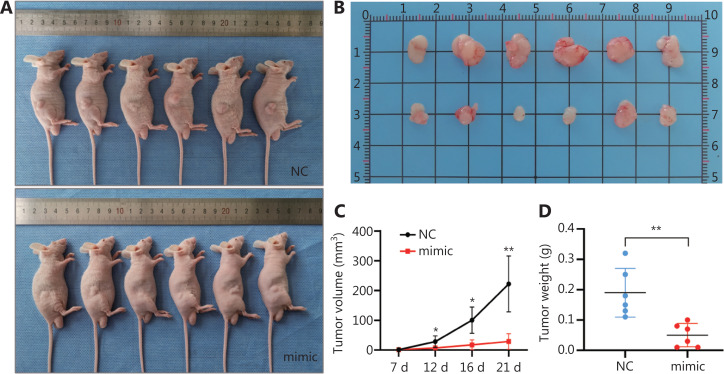
MiR-378a-3p inhibits tumorigenesis *in vivo*. (A) Image of nude mice with xenotransplantation after 3 weeks. (B) Images of tumors 3 weeks later. (C) Tumor volume, calculated and statistically analyzed over 3 weeks. (D) The mice were sacrificed for tumor weight measurement, and statistical analysis was performed after 3 weeks (*P < 0.05, **P < 0.01).

**Figure 8:**
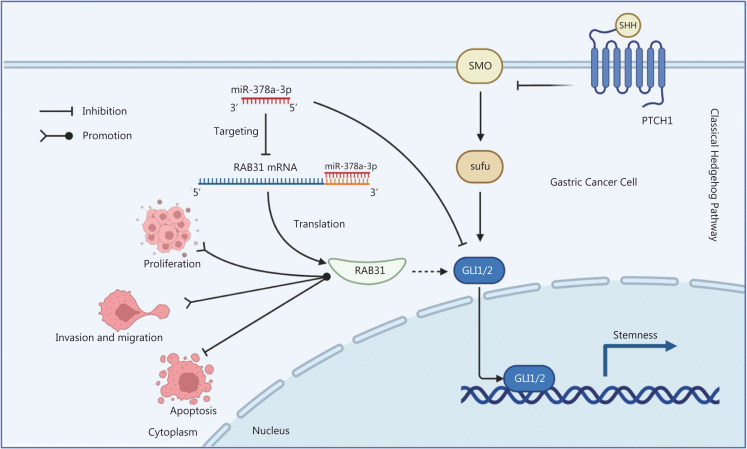
Schematic of the mechanism through which miR-378a-3p acts as a tumor suppressor in GC. MiR-378a-3p inhibits proliferation, invasion, and migration, and promotes apoptosis of GC cells by directly targeting the 3′-UTR of RAB31 mRNA. In addition, miR-378a-3p inhibits GC stemness by downregulating GLI1/2, a key molecule in the classical hedgehog signaling pathway. This inhibitory effect may be mediated by RAB31.

To further explore whether miR-378a-3p might inhibit tumorigenesis *in vivo*, we subcutaneously injected BGC-823 cells transfected with miR-378a-3p NC or miR-378a-3p mimics into male BALB/c nude mice (**Figure 7A**). The mice were sacrificed 3 weeks later, and the tumors were removed (**Figure 7B**). Then the volumes and weights of the tumors were measured. The tumor volume [(28.750±26.195) mm^3^ *vs*. (222.083±93.874) mm^3^, *P* < 0.01] and tumor weight [(0.05±0.038) g *vs*. (0.19±0.08) g, *P* < 0.01] in the miR-378a-3p mimic group were significantly lower than those in the miR-378a-3p NC group (**Figure 7C and 7D**). Together, these results implied that miR-378a-3p inhibits tumorigenesis and acts as a cancer suppressor *in vivo*. In summary, our findings indicated that miR-378a-3p functions as a tumor suppressor in GC by directly targeting RAB31 and inhibiting GLI1/2 in the Hedgehog pathway (**Figure 8**).

## Discussion

Numerous studies have shown that most miRNAs exert regulatory effects by binding specific target genes^40–42^. In addition, miRNAs can be used as new tumor markers, and have applications including early tumor detection, efficacy monitoring and evaluation, and prognostication. However, miRNAs can also participate in the formation of tumor drug resistance, and can change the microenvironment of tumor cells and their sensitivity to chemotherapy and radiotherapy; thus, they may have potential tumor treatment effects^43,44^. We wondered whether any miRNAs might directly target RAB31 and inhibit GC progression. However, we found no corresponding reports in GC.

In this study, we first identified RAB31-targeted miRNAs through a bioinformatic analysis. Further study of the 3 candidate miRNAs indicated that miR-378a-3p had the greatest effect on the expression of RAB31. Moreover, we discovered that miR-378a-3p was clearly more downregulated in GC cell lines and tissues than corresponding controls. We also observed a significant negative correlation between the expression levels of miR-378a-3p and RAB31 mRNA. Therefore, we assumed that miR-378a-3p was a tumor suppressor in GC. Subsequently, dual-luciferase assays confirmed that miR-378a-3p directly binds the 3′-UTR of RAB31 mRNA. In brief, our study reports the first identification of RAB31 as a direct target gene of miR-378a-3p.

MiR-378a-3p, a small noncoding RNA originating from chromosome 5q32, is 22 nt in length. Most studies have reported that miR-378a-3p acts as a tumor suppressor in diverse types of cancers, such as hepatocellular carcinoma^32^, colorectal cancer^45^, esophageal squamous cell carcinoma^46^, ovarian cancer^47^, cervical cancer^48^, retinoblastoma^34^, papillary thyroid cancer^49^, breast cancer^50^, prostate cancer^51^, and glioblastoma^52^. However, miR-378a-3p has also been reported to promote the progression of Burkitt lymphoma^53^. Nevertheless, the role and intrinsic mechanism of miR-378a-3p in GC have not been fully explored. In the present study, we revealed that the overexpression of miR-378a-3p inhibited the proliferation, migration, and invasion of GC cells; promoted apoptosis; and weakened the stemness of GC cells. In addition, EMT is extensively involved in tumor initiation, invasion, and metastasis. Our results further showed that miR-378a-3p inhibited the EMT in GC, in agreement with Zhang’s results in osteosarcoma^54^. Mechanistically, the rescue experiments demonstrated that miR-378a-3p inhibited the progression of GC, partly through regulation of RAB31.

Cancer stem cells (CSCs), which are closely associated with the genesis, development, and metastasis of tumors, are considered among the reasons for the heterogeneity, metastasis, recurrence, and even drug resistance in GC^55,56^. Many studies have shown that CSCs exert stem cell-like properties through various signaling pathways, such as Wnt/β-catenin, Notch, JAK/STAT, and Hippo-Yap/TAZ^57^. In addition, our previous study has indicated that the HHSP plays a crucial role in the stemness maintenance of GCSCs^38^. Generally, CSCs have surface markers that differ from those of tumor cells, such as Nanog, CD44, SOX2, and OCT4. The expression levels of these markers are not exactly the same in different tissues and tumors^58^. Here, we speculated that abnormal expression of miR-378a-3p might affect the stemness of GCSCs. As expected, both the tumorsphere formation assays and WB assays of stemness-associated markers (Nanog, CD44, SOX-2, and OCT-4) confirmed that miR-378a-3p attenuates the stemness of GC cells. Finally, a xenograft assay in nude mice further verified the tumor-suppressive effect of miR-378a-3p *in vivo*.

## Conclusions

Overall, our findings demonstrated that miR-378a-3p, which directly targets RAB31, exerts a tumor-suppressive effect in GC. Mechanistically, it downregulates the expression of RAB31 and further inhibits GLI1/2 proteins in the Hedgehog pathway. Our findings may provide a new target for the diagnosis and treatment of GC.
