# Supplementary material for: MiR-378a-3p acts as a tumor suppressor in gastric cancer via directly targeting RAB31 and inhibiting the Hedgehog pathway proteins GLI1/2
Source: Cancer Biol Med. 2022 Oct 18;19(12):1662–82. doi: 10.20892/j.issn.2095-3941.2022.0337 (PMC9755959; doi:10.20892/j.issn.2095-3941.2022.0337)
Supplement: Supplementary file 1 [file cbm-19-1662-s001.pdf]

Supplementary materials

Table S1 Oligonucleotides and plasmids used in this study

Gene	Sense sequence (5′-3′)	Anti-sense sequence (5′-3′)
MiR-378a-3p mimic	ACUGGACUUGGAGUCAGAAGGC	CUUCUGACUCCAAGUCCAGUUU
MiR-378a-3p inhibitor	GCCUUCUGACUCCAAGUCCAGU	
MiR-23b-3p mimic	AUCACAUUGCCAGGGAUUACCAC	GGUAAUCCCUGGCAAUGUGAUUU
MiR-23b-3p inhibitor	GUGGUAAUCCCUGGCAAUGUGAU	
MiR-129-2-3p mimic	AAGCCCUUACCCCAAAAAGCAU	GCUUUUUGGGGUAAGGGCUUUU
MiR-129-2-3p inhibitor	AUGCUUUUUGGGGUAAGGGCUU	
Mimic NC	UUCUCCGAACGUGUCACGUTT	ACGUGACACGUUCGGAGAATT
Inhibitor NC	CAGUACUUUUGUGUAGUACAA	
RAB31 shRNA (target sequence)	GGAGCUCAAAGUGUGCCUUTT	AAGGCACACUUUGAGCUCCTT
RAB31 negative control	UUCUCCGAACGUGUCACGUTT	ACGUGACACGUUCGGAGAATT

Table S2 Sequences of primers used in this study

Gene	Upstream (5′-3′)	Downstream (5′-3′)
RAB31	GGGGTTGGGAAATCAAGCATC	GCCAATGAATGAAACCGTTCCT
GAPDH	GGAAGCTTGTCATCAATGGAAATC	TGATGACCCTTTTGGCTCCC
MiR-378a-3p stem ring sequence	GTCGTATCCAGTGCAGGGTCCGAGGTATTCGCACTGGATACGACGCCTTC	
MiR-378a-3p	CGCGACTGGACTTGGAGTCA	AGTGCAGGGTCCGAGGTATT
MiR-23b-3p stem ring sequence	GTCGTATCCAGTGCAGGGTCCGAGGTATTCGCACTGGATACGACGTGGTA	
MiR-23b-3p	CGATCACATTGCCAGGGAT	AGTGCAGGGTCCGAGGTATT
MiR-129-2-3p stem ring sequence	GTCGTATCCAGTGCAGGGTCCGAGGTATTCGCACTGGATACGACATGCTT	
MiR-129-2-3p	CGAAGCCCTTACCCCAAA	AGTGCAGGGTCCGAGGTATT
U6	CTCGCTTCGGCAGCACA	AACGCTTCACGAATTTGCGT

Table S3 Details of antibodies used in this study

Antibody	WB/IHC dilution	Specificity	Company	Catalog number
β-Actin	1:1,000	Mouse monoclonal	CST	#3700
RAB31	1:1,000 (WB)	Rabbit polyclonal	Abcam	ab230881
RAB31	1:500 (IHC)	Rabbit polyclonal	Abcam	ab230881
GLI1	1:2,000	Mouse monoclonal	CST	#2643
GLI2	1:1,000	Rabbit monoclonal	CST	#2585
PCNA	1:1,000	Mouse monoclonal	CST	#2586
CyclinD1	1:1,000	Rabbit monoclonal	CST	#55506
Nanog	1:2,000	Rabbit monoclonal	CST	#4903
SOX2	1:1,000	Rabbit monoclonal	CST	#3579
OCT4	1:1,000	Rabbit monoclonal	CST	#2750
CD44	1:1,000	Mouse monoclonal	CST	#3570
E-cadherin	1:1,000	Mouse monoclonal	CST	#14472
N-cadherin	1:1,000	Rabbit monoclonal	CST	#13116
Vimentin	1:1,000	Rabbit monoclonal	CST	#5741
MMP2	1:1,000	Rabbit monoclonal	CST	#40994
IgG (HRP-linked)	1:10,000	Goat	BIORIGIN	BN20601/BN20604
